# Age Patterns in Self-Reported Cognitive Impairment Among Older Latino Subgroups and Non-Latino Whites in the United States, 1997–2018: Implications for Public Health Policy

**DOI:** 10.1093/geroni/igab039

**Published:** 2021-09-25

**Authors:** Marc A Garcia, David F Warner, Catherine García, Brian Downer, Mukaila Raji

**Affiliations:** 1 Department of Sociology, Aging Studies Institute, Center for Aging and Policy Studies, Lerner Center for Public Health Promotion, Maxwell School of Citizenship & Public Affairs, Syracuse, New York, USA; 2 Department of Sociology, University of Alabama at Birmingham, Birmingham, Alabama, USA; 3 Center of Family and Demographic Research, Bowling Green State University, Bowling Green, Ohio, USA; 4 Department of Human Development & Family Science, Aging Studies Institute, Center for Aging and Policy Studies, Lerner Center for Public Health Promotion, Syracuse University, Syracuse, New York, USA; 5 Division of Rehabilitation Sciences, University of Texas Medical Branch, Galveston, Texas, USA; 6 Division of Geriatrics, Department of Internal Medicine, University of Texas Medical Branch, Galveston, Texas, USA

**Keywords:** Cognitive aging, Racial/ethnic data disaggregation, Subjective cognitive decline

## Abstract

**Background and Objectives:**

U.S. Latinos are a heterogeneous population with unique characteristics related to individual-level socioeconomic and contextual factors based on nativity status and country of origin. Population aging and greater public awareness of dementia may contribute to an increasing prevalence of self-reported cognitive impairment. However, population-level trends in self-reported cognitive impairment among Latinos are unclear and it is unknown whether there are differences among Latino subgroups. Thus, this study aims to examine heterogeneity in self-reported cognitive impairment among older U.S. Latino subgroups.

**Research Design and Methods:**

We used data from the 1997–2018 National Health Interview Survey to document age-specific patterns in self-reported cognitive impairment among U.S.-born Mexican, foreign-born Mexican, island-born Puerto Rican, foreign-born Cuban, and U.S.-born non-Latino Whites aged 60 and older. We estimated hierarchical age–period–cohort cross-classified random effects models (HAPC-CCREM) to isolate age patterns in self-reported cognitive impairment across disaggregated Latino subgroups and U.S.-born non-Latino Whites.

**Results:**

The overall prevalence of self-reported cognitive impairment increased from 6.0% in 1997 to 7.1% in 2018. This increase was evident among U.S.-born non-Latino Whites and U.S.-born and foreign-born Mexicans but not other Latino subgroups. Fully adjusted HAPC-CCREM estimates indicated that Latinos were more likely to self-report cognitive impairment than U.S-born non-Latino Whites (*b* = 0.371, *p* < .001). When disaggregated by Latino subgroup, the difference in the likelihood for self-reported cognitive impairment compared to U.S.-born non-Latino Whites was greatest for island-born Puerto Ricans (*b* = 0.598, *p* < .001) and smallest for foreign-born Cubans (*b* = 0.131, *p* > .05).

**Discussion and Implications:**

We found evidence of considerable heterogeneity in the age patterns of self-reported cognitive impairment among U.S. Latino subgroups. We also detected large differences in the likelihood for self-reported cognitive impairment between U.S. Latino subgroups compared to U.S.-born non-Latino Whites. These results underscore the importance of differentiating between unique Latino subpopulations when studying population-level trends in cognitive function.

**Translational Significance:** Our findings indicate that there are substantial differences between U.S. Latino subgroups in the prevalence of self-reported cognitive impairment compared to U.S.-born non-Latino Whites. Recognizing that the U.S. Latino population includes unique subpopulations is necessary to gain a better understanding of trends in self-reported cognitive impairment and other cognitive outcomes. Differentiating between U.S. Latino subpopulations is also necessary to determine if all groups are benefiting from public health efforts to prevent cognitive impairment.

Cognitive impairment is a major public health concern in the United States that affects the quality of life and independence of older adults (Centers for Disease Control and Prevention, 2019). The prevalence of cognitive impairment is higher among older U.S. Latinos than U.S.-born non-Latino Whites (hereafter, U.S.-born Whites; Alzheimer’s Association, 2021; M. A. Garcia, Downer, et al., 2020). This disparity is attributed in part to older Latinos exhibiting longer life expectancy (Arias et al., 2021; M. A. Garcia et al., 2019; M. A. Garcia, C. Garcia, et al., 2018), lower educational attainment (Flores et al., 2017; M. A. Garcia, Reyes, et al., 2020), and a higher prevalence of chronic conditions associated with cognitive impairment, such as diabetes and cardiovascular disease (C. Garcia et al., 2018; Gupta, 2021; Menke et al., 2015) than U.S.-born Whites. Future estimates of cognitive impairment are expected to be greatest among racial/ethnic groups projected to experience the highest rates of population growth (Matthews et al., 2019). This creates an urgent need for researchers to document the cognitive risk profiles of older Latinos, a group historically underrepresented in epidemiological research (George et al., 2014; González et al., 2019), and devote specific attention to the potential for differences by nativity status and country of origin among this diverse group of aging older adults.

Subjective cognitive decline (SCD) has been defined as the self-reported experience of worsening memory, difficulty concentrating, and decrease in other thinking abilities, independent of an objective cognitive assessment or clinical diagnosis (Alzheimer’s Association, 2021; Molinuevo et al., 2017). Although older adults who report SCD typically do not have low performance on cognitive tests and are able to function independently (Jessen et al., 2020), they may exhibit biomarker evidence for Alzheimer’s disease pathology (Wolfsgruber et al., 2017). Thus, self-reported cognitive decline may represent one of the earliest noticeable indicators for decreased cognitive functioning (Alzheimer’s Association, 2021; Jessen et al., 2014), as individuals who self-report cognitive decline are more likely to progress to cognitive impairment and dementia compared to those without SCD (Donovan et al., 2014; Jessen et al., 2010; Mitchell et al., 2014; Wolfsgruber et al., 2016). Missed or delayed diagnoses of cognitive decline impede the ability to identify and treat individuals; conversely, early diagnosis of cognitive decline can facilitate the identification of treatable cases, and provide timely and accurate information to such individuals (Casillas et al., 2019; Luo et al., 2018). Studies on self-reported cognitive decline may then offer an opportunity for interventions to identify and modify potentially treatable contributors to cognitive decline (Livingston et al., 2017; Mukadam et al., 2019), so that appropriate resources and prevention efforts can be tailored to minority and immigrant groups to prevent or delay the onset of disease (Dallo et al., 2021).

Few population-based studies have used a nationally representative sample to explore racial/ethnic disparities in self-reported cognitive impairment. The literature on age, period, and cohort patterns that includes older Latino adults when examining racial/ethnic disparities in self-reported cognitive impairment is particularly limited. To date, three large population data sets have been used to document racial/ethnic disparities patterns and trends of self-reported cognitive impairment.

Luo and colleagues used the U.S. National Health Interview Survey (NHIS, 1997–2015) to examine trends in self-reported cognitive impairment among adults age 60 and older across five racial/ethnic categories (Luo et al., 2018). They found the overall prevalence of self-reported cognitive impairment increased from 5.7% in 1997 to 6.7% in 2015 and that Latinos, when assessed as a pan-ethnic group, exhibited a higher prevalence of self-reported cognitive impairment across years in the study period compared to Whites (Luo et al., 2018). Moreover, they documented that 9.3% of Latinos self-reported cognitive impairment in 1997 compared to 5.2% of Whites and that the prevalence decreased to 8.7% for Latinos and increased to 6.1% for Whites in 2015 (Luo et al., 2018). More recent evidence from the NHIS (2000–2017) estimating age- and sex-adjusted prevalence of self-reported cognitive impairment between Arab American immigrants and U.S.- and foreign-born non-Latino Whites, Blacks, Asians, and Latinos among adults aged 45 and older found the prevalence of self-reported cognitive impairment was 8.2% for foreign-born Latinos (when assessed as a pan-ethnic group) and 7.3% for U.S.-born non-Latino Whites (Dallo et al., 2021).

Additional research from the National Health and Nutrition Examination Survey (1999–2014) examined age patterns and period effects of self-reported cognitive impairment among non-Latino White, non-Latino Black, and Latino adults aged 45 and older (Casillas et al., 2019). The results showed that Latinos (8.1%–11.2%) exhibited a significantly higher prevalence of self-reported cognitive impairment over the study period (except for 2003–2006) than non-Latino Whites (4.1%–6.1%). These findings varied between midlife (age 45 and older), and late-life (65 and older) Latinos, with older adults (14.8%–21.5%) exhibiting a higher burden of self-reported cognitive impairment than their younger counterparts (7.9%–11.2%). Although the age categories differ somewhat from prior studies using the NHIS, these results corroborate that older Latinos are at an increased risk of self-reported cognitive impairment compared to Whites (Casillas et al., 2019).

Finally, research using data from the Cognitive Decline module of the Behavioral Risk Factor Surveillance System (2015–2017) found that the overall prevalence of self-reported cognitive impairment among adults aged 45 and older was 11.1%, with Latinos (11.0%) and Whites (10.9%) exhibiting comparable levels of self-reported cognitive impairment (Centers for Disease Control and Prevention, 2019; Taylor et al., 2018). This research further documented that a lower percentage of Latinos than Whites (40.2% vs 46.0%) reported discussing self-reported cognitive problems with a health care professional (Centers for Disease Control and Prevention, 2019). Different instruments, measures, analyses, and age comparison groups in the population-based data sets mentioned above may have contributed to the observed differences.

Although these studies highlight important racial/ethnic differences in SCD, they examined Latinos as an aggregate *pan-ethnic* category and thus obscured complex nativity and country of origin heterogeneity (Alcántara et al., 2021; M. A. Garcia, C. Garcia, et al., 2018; Kauh et al., 2021). Latinos are a heterogeneous group with unique sociocultural characteristics based on country of origin and immigration experiences linked to political status, social acculturation, and economic incorporation. Substantial differences in education, health coverage, health behaviors, poverty, and migratory experiences may contribute to differentials in the risk of cognitive impairment among older Mexican, Puerto Rican, Cuban, and “other” Latino populations. As the U.S. Latino population becomes more diverse, it is important to consider how variation in migration patterns, socioeconomic status, sociocultural characteristics, and contextual factors influence cognitive functioning among this group.

Indeed, there is strong theoretical and empirical evidence suggesting sociocultural heterogeneity among older Latino subgroups may contribute to differences in cognitive function. First, emerging evidence suggests we should simultaneously consider intersectional racial/ethnic, nativity status, and country of origin identities to assess social stratification factors that shape health throughout the life course to more accurately describe an individuals’ lived experiences (Brown, 2018; Brown et al., 2013; Ferraro et al., 2017; M. A. Garcia, C. Garcia, et al., 2018; M. A. Garcia, Reyes, et al., 2018; Warner & Brown, 2011). Life-course research shows that sociocultural processes may operate through differential exposure to unequal social and physical environments (Dannefer, 2003; Elder et al., 2003; Ferraro et al., 2017; Treas, 2015; Xu et al., 2018) that shape exposure to potentially modifiable risk factors for dementia (Livingston et al., 2017; Mukadam et al., 2019). Exposure to socioeconomic inequality, ethnic segregation and isolation, and lack of access to high-quality health care can create significant barriers to the use of health services, particularly among the foreign-born (Angel & Berlinger, 2018; Burr et al., 2008; Gerst & Burr, 2012). Furthermore, regional differences in health care utilization, diagnosis, and treatments to slow the progression of cognitive impairment may contribute to cognitive health disparities in the United States (Livingston et al., 2017; Mukadam et al., 2019).

Second, U.S. Latinos are heterogeneous in their population composition and their health in late life may vary as a result of differences in social and cultural characteristics related to nativity status and country of origin (Fenelon et al. 2017; C. Garcia et al., 2018; M. A. Garcia, C. Garcia, et al., 2018; M. A. Garcia, Reyes, et al., 2020). In 2015, nearly half of U.S. Latino adults were foreign-born (Flores et al., 2017). In addition, while the majority of U.S. Latinos are of Mexican-origin, Latinos of Cuban and Puerto Rican descent are increasing in proportion (Flores, 2017). However, there is a lack of knowledge regarding differences in cognitive impairment by nativity status and country of origin among older U.S. Latinos. Recent findings from a cross-national study found a higher percentage of potentially modifiable risk factors for dementia among Latin American countries than comparable low-income and middle-income countries in other world regions (Mukadam et al., 2019), suggesting that Latinos in the United States who come from the Latin American region may be especially at high risk for dementia.

Third, there is substantial demographic diversity in patterns of geographic distribution among older U.S. Latinos. The geographic dispersion of Latinos in the United States is closely linked with their country of origin. The Mexican population has historically been concentrated in the Southwestern states (e.g., California, Arizona, Colorado, Texas); the Puerto Rican population mostly in the Northeast (e.g., New York, New Jersey, Massachusetts) and in Florida; and the Cuban population predominantly in Florida (Flores, 2017). U.S. state policies and social and physical differences in the environment associated with geographic diversity may play a central role in shaping the socioeconomic, psychosocial, behavioral, and physical factors which influence the longevity and cognitive health of older Latinos (Centers for Disease Control and Prevention, 2019; Kemp & Montez, 2020; Montez et al., 2019, 2020).

Fourth, research shows substantial differences among Latino subgroups by nativity status and country of origin in the prevalence of chronic conditions (e.g., diabetes, hypertension, stroke) and health behaviors (e.g., smoking, alcohol consumption) which are known factors that contribute to disparities in cognitive function (C. Garcia et al., 2018; M. A. Garcia, C. Garcia, et al., 2018; Mukadam et al., 2019). Thus, examining disaggregated Latino subpopulations that differ substantially in geographic region, poverty, education, health coverage, migratory experiences, chronic health conditions, and longevity is crucial as they highlight the effects of inequalities that may contribute to differences in the risk of cognitive impairment.

The primary aim of this study is to explore heterogeneity among older disaggregated U.S. Latino subgroups in age patterns of self-reported cognitive impairment over 21 years of data from the 1997–2018 NHIS. We structure our study similar to that of Luo et al. (2018), who assessed racial/ethnic differences in self-reported cognitive impairment among adults aged 60 and older residing in the United States. We use the term “cognitive impairment” in its broadest sense, with a recognition that—while Alzheimer's disease and related dementias (ADRD) accounts for the majority of cognitive impairment—there are other conditions such as depression and hypothyroidism (along with or without ADRD) that comprise potentially treatable causes of cognitive impairment of different severity. Data from studying the heterogeneity in patterns, trends, and correlates of cognitive impairment stratified by disaggregated U.S. Latino subgroups have the potential to inform the development of subgroup-specific public health policies aimed at slowing cognitive decline and enhancing protective factors. Given the exploratory nature of the study, we have no a priori hypotheses about differences in the rates of self-reported cognitive impairment among disaggregated U.S. Latino subgroups.

## Method

We used data from the 1997–2018 U.S. NHIS (Blewett et al., 2019). The NHIS is a cross-sectional household survey conducted annually by the National Center for Health Statistics. The NHIS sample is representative of the civilian noninstitutionalized population residing in the United States at the time of the interview and oversamples Black and Latino households in order to increase the precision of estimates for both groups. We restricted our analyses to U.S.-born non-Latino Whites, U.S.-born Mexicans, foreign-born Mexicans, island-born Puerto Ricans, and foreign-born Cubans aged 60 and older. There were too few respondents in other Latino subgroups (e.g., U.S.-born Cubans, U.S.- and foreign-born Dominicans, and Central/South Americans) to produce reliable estimates. The inclusion criterion for age was chosen as evidence suggests that more than 90% of individuals with Alzheimer’s disease symptoms do not appear until after age 60 (Centers for Disease Control and Prevention, 2016). Missing data were minimal with 5.41% of available cases incomplete. More than 90% of incomplete cases were missing on a single variable, of which body mass index (BMI; 74.24%), education (10.68%), and heart disease (6.02%) were the most common. Given the low degree of missing data and the computational demands of the hierarchical age–period–cohort cross-classified random effects models (HAPC-CCREM), we used listwise deletion. Our final analytic sample was 139,225 adults aged 60 and older.

### Measures

Our outcome measure was self-reported cognitive impairment. Following prior research using the NHIS (Bernstein & Remsburg, 2007; Dallo et al., 2021; Luo et al., 2018), we identified respondents as having self-reported cognitive impairment if they (or a proxy) responded that their daily activities were “limited in any way because of difficulty remembering or because of experiencing periods of confusion.” Proxy reports from adult household members were permitted for other household members not present at the interview as the question was asked at the family level and linked to individual responses for each sample adult (Bernstein & Remsburg, 2007; Dallo et al., 2021).

Sociodemographic characteristics were selected based on identified risk factors for cognitive impairment and prior studies (Dallo et al., 2021; Luo et al., 2018). The covariates included were: (a) age (60–64, 65–69, 70–74, 75–79, 80–84, and ≥85 years in descriptive analyses; [continuous years, winsorized at age 85, centered at 60 in multivariate analyses]); (b) sex (female = 1; male = 0); (c) marital status (married/living with partner = 1, otherwise = 0); (d) educational attainment (at least some college = 1; otherwise = 0); and (e) the ratio of total family income to the federal poverty level (<150%; 150%–249%; 250%–499%; and ≥500% FPL, all dummy coded = 1, otherwise = 0). We also controlled for chronic health conditions and BMI. Chronic health conditions included respondent self-report of physician diagnosis with a heart attack, diabetes, hypertension, coronary artery disease, and stroke; all were dummy coded yes = 1, no = 0. BMI was measured as weight in kilograms divided by height in meters squared (kg/m^2^) and we coded this into the standard categories of underweight (<18.5 kg/m^2^), normal weight (18.5–24.9 kg/m^2^), overweight (25–29.9 kg/m^2^), and obese (>30 kg/m^2^); all were dummy coded = 1, otherwise = 0. Period and birth cohort were captured with a series of dummy variables (=1, otherwise = 0); survey year denoted period (*n* = 21, 1997–2018, centered at 1997) and birth cohort divided into nine 5- or 6-year intervals (1912–1917 to 1953–1958).

### Analytic Strategy

We first calculated survey-weighted prevalence for each of the covariates across our study sample. Then, we assessed bivariate associations between U.S.-born non-Latino Whites (referent; U.S.-born Whites) and an aggregate pan-ethnic category of Latinos using Wald-adjusted χ ^2^ comparisons. We also present estimates comparing U.S.-born Whites (referent) to disaggregated subgroups of Latinos, as well as comparing within-group differences by Latino subgroups.

Given the cross-sectional nature of the NHIS data—where age, survey year (period), and birth cohort may be linear combinations, we estimated multivariate HAPC-CCREM (Yang & Land, 2013) to minimize temporal bias. These models have been used in prior studies of cognitive performance (Gunnesch-Luca & Iliescu, 2020; Luo et al., 2018; Twenge et al., 2019). HAPC-CCREM models deal with the identification problem between age, period, and cohort effects by using a mixed model framework, specifying a fixed age effect at level 1 and random period and cohort effects at level 2 such that they are not linear combinations (Reither et al., 2015; Yang & Land, 2013, pp. 191–196). Thus, we use the HAPC-CCREM models to test whether there are developmental (age) differences in self-reported cognitive impairment across nativity and country of origin groups while adjusting for the potential period and cohort effects. Period effects are changes that affect all persons simultaneously, while cohort effects reflect the unique historical and social events experienced by a given birth cohort.

A perennial concern with age–period–cohort models is the degree of data overlap between age groups, survey periods, and birth cohorts—especially when analyzing subgroups of the population. These concerns are partially allayed by the HPAC-CCREM estimation routine (Yang & Land, 2013), but researchers should assess the consistency of model estimates by changing the size of the age, period, and cohort groupings (Masters & Powers, 2020). In sensitivity analyses (not shown) we consequently tested alternate specifications of age (i.e., winsorized at 80) and cohort (i.e., 7- and 9-year classifications). Alternate specification of age resulted in statistically significant cohort variance but had no effect on the estimated survey year (period) variance. Increasing the width of cohorts resulted in a marginally significant increase in cohort variance and a decrease in survey year variance. Importantly, across all alternative specifications, the fixed effects estimates (including for disaggregated Latino subgroups) were similar in magnitude and statistical significance to those presented here.

We estimated the HAPC-CCREM in two different ways, first comparing U.S.-born Whites to an aggregate pan-ethnic category of Latinos and then by specifying disaggregated nativity and country of origin Latino subgroups. We estimated two models: Model 1 included individual-level controls for sex and marital status; Model 2 added additional individual-level controls for education, the ratio of family income to the FPL, chronic diseases, and BMI. These social and health controls are hypothesized in prior literature to explain disparities in cognitive functioning. In all models, age was captured with both linear and quadratic terms to account for the nonlinear increase in the risk of cognitive impairment with age.

All analyses were performed using Stata/MP 16.1. Descriptive analyses applied recalibrated survey weights to minimize temporal bias resulting from variations in sampling strategies each respective year (StataCorp, 2019). The HAPC-CCREM was conducted using the meqrlogit command and weighting was not available.

## Results

### Descriptive Results

Table 1 presents the weighted prevalence of all the study variables across the racial/ethnic and nativity groups of interest. U.S.-born Whites were older, more likely to be married, better educated, and had higher incomes compared to pan-ethnic Latinos. In addition, U.S.-born Whites were more likely to have had a heart attack or to have coronary artery disease, while pan-ethnic Latinos were more likely to have diabetes, to have had a stroke, or to have a higher BMI.

**Table 1.** Weighted and Survey-Adjusted Prevalence (%) of Sample Characteristics, by Aggregated Latino Pan-Ethnicity and Disaggregated Latino Subgroups. National Health Interview Survey (NHIS), 1997–2018

Variable	Disaggregated Latino subgroups						Comparisons		
	US non-Latino White (*n* = 126,921)	Pan-ethnic Latino (*n* = 12,304)	US Mexican (*n* = 4,716)	FB Mexican (*n* = 3,802)	IB Puerto Rican (*n* = 1,779)	FB Cuban (*n* = 2,007)	*p* Value^a^	*p* Value^b^	*p* Value^c^
Age, years							<.001	<.001	<.001
60–64	0.245	0.267	0.271	0.310	0.247	0.179			
65–69	0.210	0.261	0.253	0.279	0.271	0.234			
70–74	0.177	0.186	0.190	0.171	0.205	0.187			
75–79	0.152	0.139	0.136	0.127	0.132	0.181			
80–84	0.117	0.091	0.091	0.069	0.091	0.134			
≥85	0.099	0.057	0.059	0.045	0.053	0.084			
Female	0.578	0.570	0.570	0.556	0.593	0.572	.19	.18	.01
Married	0.472	0.454	0.451	0.513	0.356	0.452	.05	<.001	<.001
Some college or more	0.501	0.243	0.315	0.112	0.223	0.346	<.001	<.001	<.001
Ratio of family income to federal poverty level							<.001	<.001	<.001
<150%	0.196	0.465	0.360	0.539	0.553	0.448			
150%–249%	0.228	0.225	0.237	0.224	0.194	0.234			
250%–499%	0.334	0.215	0.259	0.186	0.185	0.192			
≥500%	0.242	0.096	0.144	0.052	0.068	0.091			
Chronic diseases									
Heart attack	0.099	0.077	0.081	0.061	0.108	0.071	<.001	<.001	<.001
Diabetes	0.159	0.306	0.324	0.321	0.343	0.182	<.001	<.001	<.001
Hypertension	0.550	0.581	0.586	0.565	0.623	0.557	<.001	<.001	<.001
Coronary artery disease	0.132	0.113	0.110	0.102	0.139	0.116	<.001	<.001	<.001
Stroke	0.073	0.072	0.077	0.061	0.096	0.052	.70	<.001	<.001
Body mass index, kg/m^2^							<.001	<.001	<.001
Underweight (<18.5)	0.023	0.011	0.008	0.012	0.014	0.013			
Normal (18.5–24.9)	0.357	0.279	0.263	0.235	0.323	0.361			
Overweight (25.0–29.9)	0.373	0.410	0.404	0.422	0.396	0.408			
Obese (≥30.0)	0.248	0.302	0.325	0.331	0.268	0.219			

*Notes*: US = U.S.-born; FB = foreign-born; IB = island-born.

^a^Comparison between non-Latino Whites and aggregated Pan-ethnic Latinos, Wald χ ^2^.

^b^Comparison among US non-Latino Whites and disaggregated Latino subgroups, Wald χ ^2^.

^c^Comparison within disaggregated Latino subgroups, Wald χ ^2^.

Looking at differences among disaggregated Latino groups we see all study variables significantly differ, indicating considerable heterogeneity. Foreign-born Mexicans were the youngest group ($\bar{x}$ = 69.23), while foreign-born Cubans were the oldest ($\bar{x}$ = 72.40). Island-born Puerto Ricans had a higher proportion of females (59.31%) and were least likely to be married (35.55%), while foreign-born Mexicans had a lower proportion of females (55.62%) and were most likely to be married (51.30%). U.S.-born Mexicans had the highest incomes, while foreign-born Mexicans and island-born Puerto Ricans had the lowest. Across chronic diseases, island-born Puerto Ricans had the highest prevalence rates, but the group with the lowest rate differed by disease. Foreign-born Mexicans were the most likely to be obese (33.06%), while foreign-born Cubans were the least (21.91%).

The overall prevalence of self-reported cognitive impairment increased slightly between 1997 and 2018, from 5.96% to 7.06% (*p* < .001 for linear trend; not shown). Disaggregating by nativity status and country of origin, we found little evidence of differences across groups (see Supplementary Table 1). Consistent with the overall trend, however, we found a significant increase over time for U.S.-born Whites [(*p* < .001 for trend) and (marginally) for pan-ethnic Latinos (*p* = .066)]. Among the disaggregated nativity and country of origin Latino subgroups, we found a significant increase over time for U.S.-born Mexicans (*p* = .039) but there were no discernible time trends in self-reported cognitive impairment for foreign-born Mexicans, island-born Puerto Ricans, or foreign-born Cubans. Cross-comparisons between U.S.-born Whites and each of the disaggregated Latino groups did not show any statistically significant difference in the linear trends. However, there was some suggestion that the time trend for U.S.-born Whites and foreign-born Mexicans did significantly differ when a nonlinear (quadratic) trend was specified for foreign-born Mexicans (*p = .*008; not shown). Overall, there do not appear to be any differences in the linear rate of increase in self-reported cognitive impairment across Latino subgroups between 1997 and 2018 (see also Luo et al., 2018).

**Figure 1.**
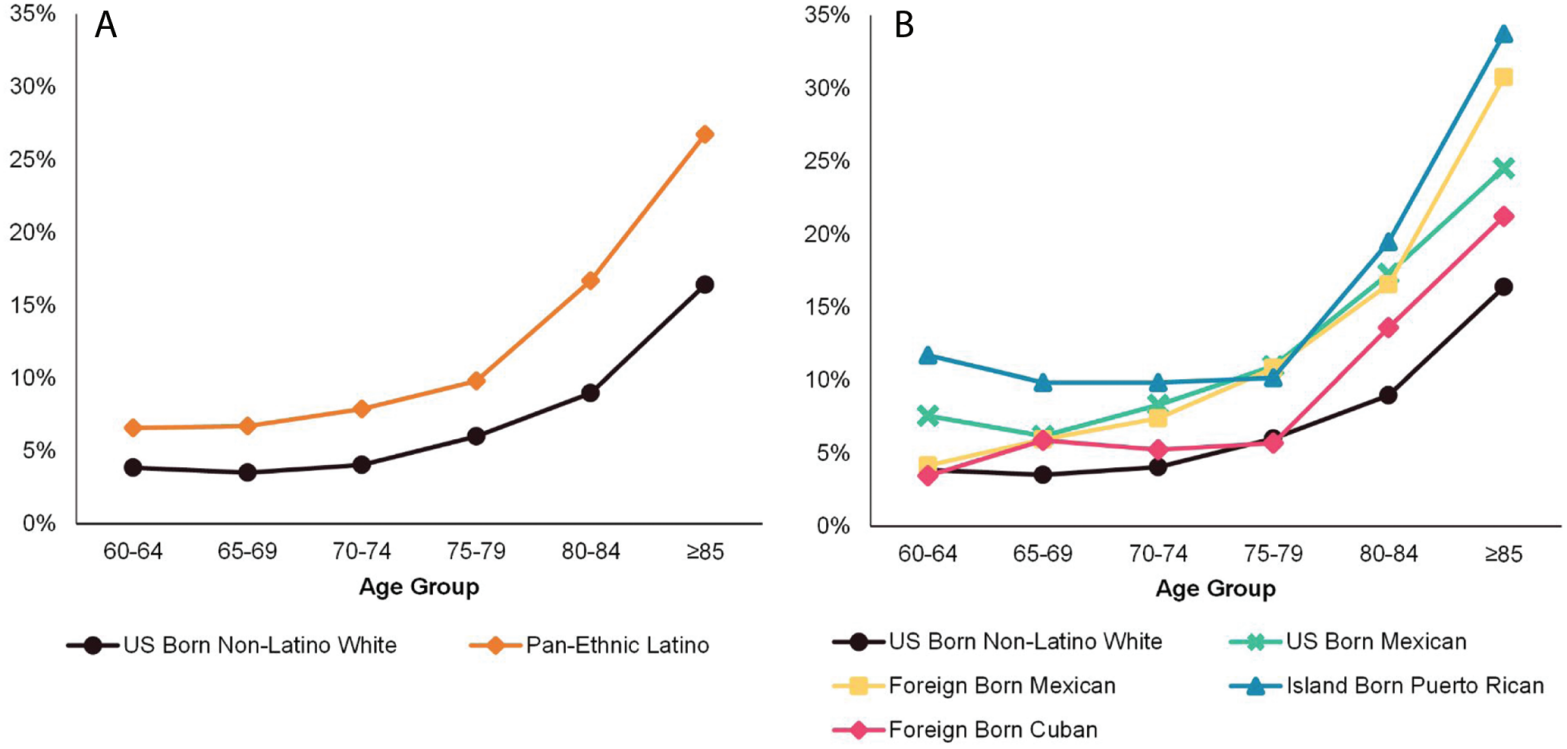
Weighted and survey-adjusted prevalence (%) of self-reported cognitive impairment: (A) Age group by Latino pan-ethnicity and (B) Age group by disaggregated nativity and country-of-origin Latino subgroups. National Health Interview Survey, 1997–2018.

As displayed in Figure 1, examining the age patterns in the prevalence of self-reported cognitive impairment revealed a significant nonlinear increase with age for all disaggregated nativity and country of origin groups (*p* < .001 for all groups; tests not shown). This age pattern was also evident when specifying age as either a linear or categorical measure (not shown). The prevalence of self-reported cognitive impairment increased markedly after age 80 for all groups. Across age, U.S.-born Whites had lower rates of self-reported cognitive impairment than aggregated pan-ethnic Latinos (see Figure 1A; *p* < .001); however, the difference in the rates with age was not significantly different between the two (not shown). Examining differences between U.S.-born Whites and the disaggregated nativity and country of origin Latino subgroups (see Figure 1B; results not shown) indicated that U.S.-born Mexicans (*p < .*001), foreign-born Mexicans (*p < .*001), island-born Puerto Ricans (*p* < .001), and foreign-born Cubans (*p* = .033) had significantly higher levels of self-reported cognitive impairment on average than U.S.-born Whites. Similar to the comparison of the age pattern with aggregated pan-ethnic Latinos, the increase with age did not differ from that of U.S.-born Whites for either U.S.-born Mexicans or foreign-born Cubans. However, island-born Puerto Ricans (*p* = .019) exhibited a larger linear increase with age and foreign-born Mexicans exhibited a larger and nonlinear increase with age (*p* < .001) compared to U.S.-born Whites.

**Figure 2.**
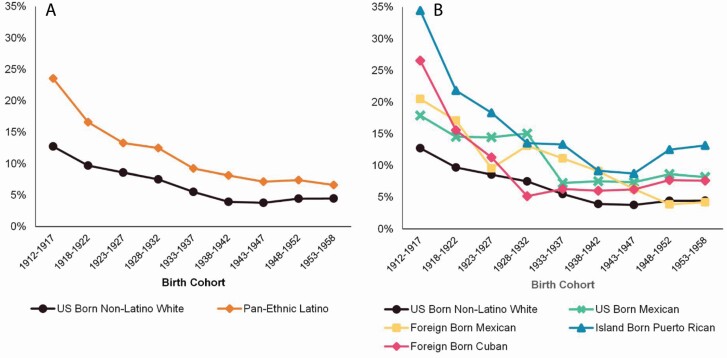
Weighted and survey-adjusted prevalence (%) of self-reported cognitive impairment: (A) Birth cohort by Latino pan-ethnicity and (B) Birth cohort by disaggregated nativity and country-of-origin Latino subgroups. National Health Interview Survey, 1997–2018.

Birth cohort patterns in self-reported cognitive impairment were, as expected, largely the inverse of those by age (see Figure 2). Earlier birth cohorts had higher rates of self-reported cognitive impairment, and this declined in a nonlinear fashion for each later-born cohort. Across cohorts, U.S.-born Whites had lower rates of self-reported cognitive impairment than aggregated pan-ethnic Latinos (see Figure 2A; *p* < .001), and the rate of change across cohorts did not differ between the two (results not shown). We found similar nonlinear cohort trends when examining the disaggregated Latino subgroups (see Figure 2B). Tests comparing the various cohort differences indicated that all disaggregated nativity and country of origin Latino subgroups differed from U.S.-born Whites overall and in the nonlinear rate of change—except for U.S.-born Mexicans whose rate of change across cohorts did not differ from that of U.S.-born Whites (tests not shown). We found similar patterns and comparisons when specifying cohort as either a linear or categorical measure (not shown).

### Multivariate Results

The multivariate HAPC-CCREM models adjusting for the potential period and cohort influences are presented in Table 2. Models 1 and 2 compared U.S.-born Whites with aggregated pan-ethnic Latinos and Models 3 and 4 compared U.S.-born Whites with disaggregated Latino nativity and country of origin subgroups. In both cases, the first model (Models 1 and 3) presents a baseline model that only includes basic demographics, while the second model (Models 2 and 4) includes social and health covariates hypothesized in the literature to be explanations for disparities in cognitive functioning.

**Table 2.** Hierarchical Age–Period–Cohort Cross-Classified Model Results for Factors Associated With Self-Reported Cognitive Impairment, by Aggregated Latino Pan-Ethnicity and Disaggregated Latino Subgroups. National Health Interview Survey (NHIS), 1997–2018

Variable	US non-Latino Whites compared to aggregated pan-ethnic Latinos		US non-Latino Whites compared to disaggregated Latino subgroups^c^	
	Model 1	Model 2	Model 3	Model 4
	*B* (*SE*)	*B* (*SE*)	*B* (*SE*)	*B* (*SE*)
Fixed effects				
Intercept	−2.871 (0.034)***	−2.014 (0.085)***	−2.876 (0.055)***	−2.014 (0.084)***
Age^a^	−0.051 (0.007)***	−0.073 (0.000)***	−0.051 (0.007)***	−0.073 (0.007)***
Age^2^	0.004 (0.000)***	0.005 (0.000)***	0.004 (0.000)***	0.005 (0.000)***
Sex				
Male	Ref.	Ref.	Ref.	Ref.
Female	−0.115 (0.024)***	−0.125 (0.025)***	−0.116 (0.025)***	−0.126 (0.025)***
Marital status				
Not married	Ref.	Ref.	Ref.	Ref.
Married	−0.466 (0.025)***	−0.275 (0.027)***	−0.461 (0.025)***	−0.270 (0.027)***
Race/ethnicity/nativity				
Non-Latino White	Ref.	Ref.	Ref.	Ref.
Pan-ethnic Latino	0.627 (0.034)***	0.371 (0.036)***	—	—
US Mexican	—	—	0.666 (0.052)***	0.445 (0.054)***
FB Mexican	—	—	0.576 (0.061)***	0.265 (0.063)***
IB Puerto Rican	—	—	0.958 (0.074)***	0.598 (0.077)***
FB Cuban	—	—	0.259 (0.087)**	0.131 (0.089)
Education				
High school or less		Ref.		Ref.
Some college or more		−0.136 (0.026)***		−0.136 (0.026)***
Ratio of family income to federal poverty level				
<150%		Ref.		Ref.
150%–249%		−0.396 (0.030)***		−0.397 (0.030)***
250%–499%		−0.605 (0.031)***		−0.608 (0.031)***
≥500%		−0.972 (0.044)***		−0.976 (0.044)***
Chronic diseases				
Heart attack		0.215 (0.037)***		0.212 (0.037)***
Diabetes		0.351 (0.029)***		0.347 (0.029)***
Hypertension		0.114 (0.025)***		0.113 (0.025)***
Coronary artery disease		0.258 (0.034)***		0.259 (0.034)***
Stroke		1.275 (0.029)***		1.272 (0.029)***
Body mass index, kg/m^2^				
Underweight (<18.5)		Ref.		Ref.
Normal (18.5–24.9)		−0.507 (0.059)***		−0.506 (0.059)***
Overweight (25.0–29.9)		−0.757 (0.061)***		−0.756 (0.061)***
Obese (≥3.0)		−0.588 (0.063)***		−0.587 (0.063)***
Random effects				
Survey period^b^	0.012 (0.005)*	0.017 (0.007)**	0.011 (0.005)**	0.017 (0.007)**
1997	0.018 (0.048)	−0.003 (0.051)	0.020 (0.048)	−0.002 (0.051)
1998	−0.138 (0.052)**	−0.172 (0.056)**	−0.139 (0.052)**	−0.175 (0.056)**
1999	−0.230 (0.054)***	−0.232 (0.058)***	−0.228 (0.054)***	−0.233 (0.058)***
2000	−0.123 (0.052)**	−0.144 (0.055)**	−0.121 (0.052)**	−0.144 (0.055)**
2001	−0.093 (0.051)^†^	−0.108 (0.055)*	−0.093 (0.051)^†^	−0.108 (0.055)*
2002	−0.038 (0.051)	−0.063 (0.054)	−0.036 (0.051)	−0.062 (0.055)
2003	−0.035 (0.052)	−0.029 (0.055)	−0.034 (0.052)	−0.028 (0.055)
2004	−0.028 (0.050)	−0.085 (0.053)	−0.027 (0.050)	−0.084 (0.054)
2005	−0.013 (0.050)	−0.035 (0.053)	−0.012 (0.050)	−0.034 (0.053)
2006	−0.084 (0.057)	−0.121 (0.062)*	−0.086 (0.057)	−0.122 (0.062)*
2007	−0.049 (0.055)	0.066 (0.059)	0.048 (0.055)	0.066 (0.059)
2008	−0.047 (0.057)	−0.071 (0.061)	−0.049 (0.057)	−0.072 (0.061)
2009	0.002 (0.052)	−0.005 (0.055)	0.003 (0.052)	−0.004 (0.055)
2010	0.046 (0.051)	0.052 (0.055)	0.044 (0.051)	−0.052 (0.055)
2011	0.083 (0.046)^†^	0.082 (0.047)^†^	0.081 (0.046)^†^	0.081 (0.049)^†^
2012	0.048 (0.046)	0.039 (0.048)	0.046 (0.045)	0.038 (0.048)
2013	0.056 (0.045)	0.067 (0.048)	0.054 (0.045)	0.066 (0.048)
2014	0.037 (0.044)	0.071 (0.046)	0.038 (0.044)	0.073 (0.046)
2015	0.075 (0.043)^†^	0.101 (0.046)*	0.077 (0.043)^†^	0.103 (0.046)*
2016	0.097 (0.042)*	0.149 (0.044)***	0.095 (0.042)*	0.149 (0.044)***
2017	0.176 (0.044)***	0.248 (0.047)***	0.176 (0.044)***	0.249 (0.047)***
2018	0.129 (0.045)***	0.209 (0.048)***	0.158 (0.045)***	0.210 (0.048)***
Birth cohort^b^	0.002 (0.002)	0.003 (0.003)	0.002 (0.003)	0.003 (0.003)
1912–1917	0.003 (0.033)	0.010 (0.036)	−0.023 (0.030)	0.011 (0.036)
1918–1922	−0.023 (0.030)	−0.024 (0.033)	−0.023 (0.030)	−0.025 (0.033)
1923–1927	0.002 (0.027)	−0.001 (0.030)	−0.002 (0.027)	−0.005 (0.030)
1928–1932	−0.038 (0.025)	−0.045 (0.027)^†^	−0.037 (0.025)	−0.046 (0.027)^†^
1933–1937	−0.011 (0.027)	−0.019 (0.029)	−0.010 (0.027)	−0.019 (0.029)
1938–1942	−0.028 (0.028)	−0.027 (0.030)	−0.028 (0.028)	−0.027 (0.030)
1943–1947	−0.000 (0.029)	−0.005 (0.030)	−0.000 (0.029)	−0.005 (0.031)
1948–1952	0.083 (0.030)**	0.097 (0.033)**	0.083 (0.035)**	0.097 (0.033)**
1953–1958	−0.014 (0.035)	0.012 (0.038)	0.015 (0.035)	0.012 (0.038)

*Notes*: US = U.S.-born; FB = foreign-born; IB = island-born.

^a^Centered at 60.

^b^Variance estimate.

^c^See text for discussion of tests for differences among nativity and country of origin Latino subgroups.

^†^ *p* < .10. *p < .05. **p < .01. ***p < .001 (two-tailed tests).

Examining the models comparing U.S.-born Whites with aggregated pan-ethnic Latinos, we find a strong and nonlinear increase in self-reported cognitive impairment with age even after accounting for period and cohort. Net of protective effects of being female and married, older Latinos were about 65% more likely to self-report cognitive impairment (β = 0.627, *p* < .001; Table 2, Model 1). The variance by survey year is statistically significant (τ = 0.012, *p* = .021) with the estimated average effects for the number of survey years statistically significant. However, after controlling for age and other individual measures, there is no statistically significant variance by cohort (τ = 0.002, *p* = .363). We note that the estimated average effect for the 1948–1952 birth cohort did suggest that older adults from this cohort had slightly higher rates of self-reported cognitive impairment (β = 0.083, *p* = .006) even as the overall cohort variance was not significant.

Including the social and health covariates in Model 2 reduced the odds of older Latinos reporting self-reported cognitive impairment; nevertheless, older Latinos remained 59% more likely to self-report cognitive impairment (β = 0.371, *p* < .001). Controlling for education, income, chronic diseases, and BMI had little substantive effect on the age, sex, and marital status estimates described above. As expected, education and income were negatively associated with self-reported cognitive impairment, whereas all the chronic diseases were associated with greater odds of self-reported cognitive impairment—particularly stroke. Higher BMI was generally protective (compared to being underweight). The inclusion of these factors did not appreciably reduce the period or (nonsignificant) cohort variance estimates.

Turning to the models specifying disaggregated nativity and country of origin Latino subgroups, we observe significant heterogeneity among Latinos (Table 2, Model 3). Compared to U.S.-born Whites, all disaggregated nativity and country of origin Latino subgroups were more likely to self-report cognitive impairment, with the greatest difference for island-born Puerto Ricans (β = 0.958, *p* < .001), followed by U.S.-born Mexicans (β = 0.666, *p* < .001), foreign-born Mexicans (β = 0.576, *p* < .001), and the smallest difference for foreign-born Cubans (β = 0.259, *p* = .002). In addition, all disaggregated nativity and country of origin Latino subgroups were significantly different from one another (at least *p* < .01; not shown). The estimated effects of age, sex, and marital status—as well as the period and cohort variance components and random effects—were nearly identical to those in Model 1.

Adding social and health covariates (Table 2, Model 4), reduced the magnitude of the differences between U.S.-born Whites and all disaggregated nativity and country of origin subgroups by 30%–50%. All disaggregated nativity and country of origin subgroups were significantly different (*p* < .001) from U.S.-born Whites, except for foreign-born Cubans (*p* = .142). However, the inclusion of these covariates did render some of the differences in the odds of self-reported cognitive impairment between disaggregated nativity and country of origin Latino subgroups statistically nonsignificant. Specifically, U.S.-born Mexicans were not significantly different from island-born Puerto Ricans (*p* = .092; not shown); foreign-born Cubans were not different from foreign-born Mexicans (*p* = .210). This suggests that accounting for differences in individual-level social and health characteristics reduces ethnic differences among disaggregated Latinos, though nativity differences remained—as each U.S.-born group (including island-born Puerto Ricans) was statistically different from each foreign-born group. Again, the estimated effects of the covariates, the period and cohort variance components, and the random effects were substantively similar to those from Model 2 discussed above.

In supplemental analysis, we tested whether the estimated effect of age on self-reported cognitive impairment differed between U.S.-born Whites and each nativity/country of origin Latino subgroup. The results in Table 2 constrain the estimated age effects to be the same for all groups. Using Model 4 of Table 2 as a starting point, we specified a series of interaction terms between each nativity/country of origin Latino group with the age and age-squared terms and then performed a joint test of statistical significance to determine whether group-specific age effects were warranted. The results of this model indicated that only for older foreign-born Mexican adults were the joint age effects significantly different from U.S.-born Whites (Wald χ ^2^ = 15.36, *p* < .001); none of the other interaction terms were jointly significant (not shown). Further exploration showed that the estimated age effect for foreign-born Mexicans did not differ from that of U.S.-born Whites through the early 70s (ie, the estimated odds, of impairment are almost identical), after which foreign-born Mexicans were significantly more likely to self-report cognitive impairment and this difference slightly widened with age (not shown). Although this adds a bit of nuance to the results presented in Table 2, the general conclusion remains that all disaggregated Latino nativity and country of origin subgroups are more likely to self-report cognitive impairment with age than U.S.-born Whites and that social and health differences between groups contribute to such differences between U.S.-born and foreign-born subgroups.

## Discussion

Our study identifies several key findings regarding self-reported cognitive impairment among disaggregated Latino populations aged 60 and older residing in the United States. First, the prevalence of self-reported cognitive impairment increased with age, and markedly so after the age of 80, for U.S.-born non-Latino Whites and all disaggregated Latino subgroups in our study. This age patterning is consistent with the increase in the incidence of Alzheimer’s disease and related dementias after age 80 (Rajan et al., 2019; Tom et al., 2015).

Second, we found higher rates of self-reported cognitive impairment among all disaggregated Latino subgroups compared to U.S.-born Whites. These findings build on prior studies documenting cognitive disparities between Latinos (when assessed as a pan-ethnic group) and U.S.-born Whites that did not consider nativity and country of origin. Specifically, we document island-born Puerto Ricans exhibit the greatest difference in the likelihood for self-reported cognitive impairment relative to U.S.-born Whites, whereas foreign-born Cubans exhibit the smallest difference compared to U.S.-born Whites. Future research might consider how educational improvements among later-born Latino birth cohorts affect self-reported cognitive impairment as a potential mechanistic pathway shaping decreases in self-reported cognitive impairment across birth cohorts.

Third, after adjustment for known social and health confounding measures, our HAPC-CCREM estimates show that older Latinos were more likely to self-report cognitive impairment compared to U.S.-born Whites. All disaggregated nativity and country of origin Latino subgroups (except foreign-born Cubans) exhibited a higher likelihood of self-reported cognitive impairment than U.S.-born Whites. Importantly, in the fully adjusted model, each of the disaggregated Latino subgroups had rates of self-reported cognitive impairment significantly different from one another. The remaining observed differences between Latino subgroups may reflect heterogeneity in sociocultural, socioeconomic, and other health characteristics of older Latino adults associated with nativity status and country of origin (Abrams et al., 2021; Arroyo-Johnson et al., 2016; Daviglus et al., 2012; C. Garcia et al., 2018; M. A. Garcia, Downer, et al., 2021; M. A. Garcia, C. Garcia, et al., 2018; Pabon-Nau et al., 2010) that we are unable to measure in the NHIS.

Finally, we note there was some evidence that the increase in self-reported cognitive impairment across the study period (1997–2018) was significantly greater for U.S.-born and foreign-born Mexicans compared to U.S.-born Whites. These differences in the prevalence rates by year further reinforce the importance of considering nativity status and country of origin among Latinos when documenting population-level trends in cognitive outcomes—especially because there was no apparent difference between U.S.-born Whites and aggregated pan-ethnic Latinos. Intensifying sociopolitical scrutiny experienced by both U.S.-born and foreign-born Mexicans during our study period might be a plausible explanation. Such scrutiny can have deleterious mental health consequences that may fuel higher rates of depression and anxiety (C. García, M. A. Garcia, et al., 2019). Decreased access to adequate care for diabetes and hypertension—stroke-causing diseases highly prevalent in Latinos, suboptimal mental health services, coupled with understandable medical mistrust among Latinos, may also contribute to a subsequent heightened risk for factors related to cognitive impairment. This is especially likely to be the case in Latino-heavy states, such as Texas, that have failed to expand Medicaid under the 2010 Affordable Care Act. Although our study focuses on differences by nativity and country of origin, the implications of sociopolitical scrutiny may also differ by generation—with the U.S.-born children of immigrants perhaps more at risk for such deleterious effects and heightened cognitive impairment than those of U.S.-born parents. Future research should consider such heterogeneity in cognitive impairment trends among other major racial/ethnic groups, including U.S. Whites as recent evidence indicates large variation in health disparities within the White population, in part due to declines in Whites of Western European descent and increases in Whites of Eastern European and Middle Eastern descent (Kauh et al., 2021; Read et al., 2021).

Our study is not without limitations. First, self-reported cognition is subject to recall and reporting bias. Although public knowledge about dementia is generally low (Cahill et al., 2015), recent public health campaigns may be contributing to increased knowledge about the early signs and symptoms of Alzheimer’s disease (Cations et al., 2018). This may make people more aware of changes in memory or cognition that negatively affect daily life. Conversely, some older adults may not recognize or may deny that they are experiencing changes in cognitive functioning (Chung & Man, 2009; Roberts et al., 2009). Second, recognizing the highly stigmatized perceptions of mental health among Latino populations, respondents may have been reluctant to provide an accurate response regarding cognitive impairment. Third, the NHIS does not include measurement of previous self-reported cognitive impairment; therefore, respondents may have experienced cognitive decline prior to being administered the survey and thus supplied unreliable reports.

Fourth, our analytic sample is not representative of other Latino subgroups (e.g., U.S.-born Cubans, U.S.- and foreign-born Dominicans, and U.S.- and foreign-born Central/South Americans). The NHIS asked individuals who identify as Latino for their national origin/ancestry, which includes Dominicans, Central/South Americans, multiple heritage Latinos, and other Latino/Hispanic/Spanish individuals whose country of origin is not specified. We excluded these individuals from our analysis due to small sample sizes over the observed period; however, we recognize these groups contribute to the heterogeneity within the overall Latino population that may have been captured in prior studies using aggregate pan-ethnic categories. Supplemental analyses including all self-identified Latinos in the aggregate pan-ethnic Latino group showed similar patterns and findings to those presented here (not shown).

 Fifth, the NHIS sampling strategy does not include survey administration in long-term care facilities. Studies show that older adults residing in long-term care facilities often exhibit an increased prevalence of cognitive impairment compared to community-dwelling older adults. Thus, the higher rates of self-reported cognitive impairment we identify among disaggregated Latino subgroups may be conservative.

Finally, we recognize that modern age–period–cohort approaches have received some critiques in the literature (Bell & Jones, 2014; Masters & Powers, 2020; Reither et al., 2015), largely surrounding model assumptions. The advantage of the HAPC-CCREM approach is that the model assumptions are mechanical, that is, less influenced by investigator specifications (Masters & Powers, 2020), rather than requiring strong parameter constraints as was common with earlier approaches (Reither et al., 2015). A primary area where investigator decisions may matter is in the size of the different age–period–cohort groupings. As we noted above, our sensitivity analyses using alternate age and cohort grouping sizes demonstrate that our results were robust to different specifications.

In conclusion, analysis from our investigation showed that between 1997 and 2018, the prevalence of self-reported cognitive impairment increased among U.S.-born Whites and Latinos, largely driven by U.S.-born Mexicans, aged 60 and older. The overall prevalence of self-reported cognitive impairment increased across the study period, as we found that earlier-born cohorts exhibited higher prevalence rates of self-reported cognitive impairment compared to later-born cohorts. Our multivariate analyses make clear that the prevalence of self-reported cognitive impairment is higher among Latinos compared to U.S.-born Whites, but importantly there are significant differences among all nativity and country of origin Latino subgroups. These differences likely reflect—at least in part—unmeasured differential exposure to one or more of the nine policy-actionable and potentially modifiable dementia risk factors (less childhood education, midlife hearing loss, hypertension, and obesity, and later-life smoking, depression, physical inactivity, social isolation, and diabetes). These factors are also largely influenced by differential exposure (e.g., by nativity status and country of origin) to myriad social, economic, political, behavioral, and environmental determinants of brain health. Furthermore, the differences we continue to observe among nativity and country of origin Latino subgroups, and in comparison to U.S.-born non-Latino Whites, may reflect differential environmental exposures given the growing evidence that urban air pollution and indoor air pollution (i.e., combustion of solid cooking fuels in low- and middle-income countries) affect cognitive health (Ailshire & Clarke, 2015; Ailshire & Crimmins, 2014; Kulick et al., 2020; Saenz, 2021; Saenz et al., 2018, 2021). Future research should aim to further investigate differences in such exposure across groups by, for example, matching respondents on urbanicity to account for factors associated with cognition not captured in this study.

Overall, our findings underscore the importance of disaggregating pan-ethnic group data to highlight critical within-group heterogeneity, in terms of nativity and country of origin, among Latinos when evaluating cognitive health (Alcántara et al., 2021; M. A. Garcia, C. Garcia, et al., 2018; Kauh et al., 2021). Additionally, the findings stress the need for the development and implementation of targeted interventions anchored in data-driven culturally relevant treatment options for older Latino subgroups (Alcántara et al., 2021; M. A. Garcia, C. Garcia, et al., 2018; Kauh et al., 2021). Minimizing the deleterious effects of cognitive impairment among the largest racial/ethnic group in the United States requires concerted efforts within medical/public health institutions to ameliorate inequities in cognitive health. Public health efforts to minimize the expected burden of Alzheimer’s disease and related dementias need to consider the changing demographic shifts that contextualize the findings within our investigation.

## Supplementary Material

igab039_suppl_Supplementary_Materials

## Data Availability

The NHIS is publicly available but individuals must register for an account at https://nhis.ipums.org/nhis/ihis.org and agree to the terms to access the data.

## Code Availability

Code is available to users upon request.
