# Supplementary material for: Age Patterns in Self-Reported Cognitive Impairment Among Older Latino Subgroups and Non-Latino Whites in the United States, 1997–2018: Implications for Public Health Policy
Source: Innov Aging. 2021 Sep 25;5(4):igab039. doi: 10.1093/geroni/igab039 (PMC8670720; doi:10.1093/geroni/igab039)
Supplement: igab039_suppl_Supplementary_Materials [file igab039_suppl_supplementary_materials.docx]

**Supplementary Table 1.** Weighted and Survey-Adjusted Prevalence (%) of Cognitive Impairment across Survey Years, by Aggregated Latino Pan-ethnicity and Disaggregated Latino Subgroups. National Health Interview Survey (NHIS), 1997-2018.

|  |  | | | Disaggregated Latino Subgroups | | | |
| --- | --- | --- | --- | --- | --- | --- | --- |
| Year |  | US Non-Latino White  (n= 126,921) | Pan-Ethnic Latino  (n=12,304) | US Mexican  (n=4,716) | FB Mexican  (n=3,802) | IB Puerto Rican  (n=1,779) | FB Cuban  (n=2,007) |
| 1997 |  | .057 | .119 | .091 | .159 | .173 | .080 |
| 1998 |  | .051 | .079 | .070 | .081 | .125 | .042 |
| 1999 |  | .043 | .061 | .053 | .063 | .103 | .043 |
| 2000 |  | .049 | .079 | .056 | .084 | .129 | .082 |
| 2001 |  | .053 | .087 | .072 | .034 | .204 | .090 |
| 2002 |  | .056 | .086 | .110 | .061 | .160 | .022 |
| 2003 |  | .058 | .065 | .070 | .098 | .022 | .056 |
| 2004 |  | .057 | .098 | .096 | .094 | .094 | .112 |
| 2005 |  | .059 | .092 | .125 | .068 | .071 | .084 |
| 2006 |  | .054 | .078 | .067 | .057 | .142 | .064 |
| 2007 |  | .065 | .098 | .107 | .039 | .172 | .068 |
| 2008 |  | .056 | .087 | .106 | .055 | .100 | .087 |
| 2009 |  | .062 | .078 | .088 | .096 | .034 | .057 |
| 2010 |  | .063 | .100 | .091 | .085 | .157 | .082 |
| 2011 |  | .068 | .092 | .122 | .045 | .127 | .065 |
| 2012 |  | .061 | .109 | .089 | .092 | .201 | .088 |
| 2013 |  | .067 | .095 | .103 | .056 | .153 | .069 |
| 2014 |  | .062 | .090 | .114 | .076 | .076 | .080 |
| 2015 |  | .061 | .095 | .092 | .075 | .135 | .095 |
| 2016 |  | .062 | .108 | .117 | .082 | .144 | .109 |
| 2017 |  | .072 | .115 | .143 | .123 | .058 | .056 |
| 2018 |  | .068 | .099 | .082 | .096 | .140 | .112 |
| Test for Linear Trend^a,b^ | | *p* <.001 | *p* =.066 | *p* =.039 | *p* >.10 | *p* > .10 | *p* >.10 |
| *Notes.* US = U.S.-Born; FB= Foreign-Born; IB = Island-Born.  ^a^ Test for linear trend performed using survey-adjusted logistic regression model; ^b^ No significance difference in linear trends between aggregated pan-ethnic Latino or disaggregated Latino subgroups and US non-Latino Whites. | | | | | | | |
